# Inductive specification and axonal orientation of spinal neurons mediated by divergent bone morphogenetic protein signaling pathways

**DOI:** 10.1186/1749-8104-6-36

**Published:** 2011-11-15

**Authors:** Jeanette C Perron, Jane Dodd

**Affiliations:** 1 Department of Physiology and Cellular Biophysics, Columbia University, 630 West 168th Street (BB1103), New York, NY 10032, USA; 2 Department of Neuroscience, Columbia University, 630 West 168th Street (BB1103), New York, NY 10032, USA

## Abstract

**Background:**

Bone morphogenetic protein (BMP)7 evokes both inductive and axon orienting responses in dorsal interneurons (dI neurons) in the developing spinal cord. These events occur sequentially during the development of spinal neurons but in these and other cell types such inductive and acute chemotactic responses occur concurrently, highlighting the requirement for divergent intracellular signaling. Both type I and type II BMP receptor subtypes have been implicated selectively in orienting responses but it remains unclear how, in a given cell, divergence occurs. We have examined the mechanisms by which disparate BMP7 activities are generated in dorsal spinal neurons.

**Results:**

We show that widely different threshold concentrations of BMP7 are required to elicit the divergent inductive and axon orienting responses. Type I BMP receptor kinase activity is required for activation of pSmad signaling and induction of dI character by BMP7, a high threshold response. In contrast, neither type I BMP receptor kinase activity nor Smad1/5/8 phosphorylation is involved in the low threshold orienting responses of dI axons to BMP7. Instead, BMP7-evoked axonal repulsion and growth cone collapse are dependent on phosphoinositide-3-kinase (PI3K) activation, plausibly through type II receptor signaling. BMP7 stimulates PI3K-dependent signaling in dI neurons. BMP6, which evokes neural induction but does not have orienting activity, activates Smad signaling but does not stimulate PI3K.

**Conclusions:**

Divergent signaling through pSmad-dependent and PI3K-dependent (Smad-independent) mechanisms mediates the inductive and orienting responses of dI neurons to BMP7. A model is proposed whereby selective engagement of BMP receptor subunits underlies choice of signaling pathway.

## Background

Factors first identified as inductive signals that regulate cell fate and tissue organization have recently been shown to have crucial roles in acute activities such as growth cone guidance and axon path finding [1]. This principle emerged from studies of the developmental actions of fibroblast growth factors and bone morphogenetic proteins (BMPs) [2-4], and has been shown more recently also to apply to Wnt [5,6] and Hh [7] signaling. These observations pose the question of how distinctive developmental activities can be generated by the same ligand. In principle, a number of strategies might achieve such a dichotomy: different presentation of the ligand and/or mechanisms of selective receptor engagement could activate distinct intracellular pathways. The initiation of parallel or divergent signaling cascades presumably lies at the heart of distinct cellular events. But where and how such signaling pathways diverge remains unclear.

BMPs trigger long-term inductive signaling events that involve gene transcription and/or the acute cellular responses of chemotaxis and axon orientation, in both neurons and non-neuronal cells [3,8]. Instances in which long-term and acute responses to the same BMP can occur concurrently in a single cell, illustrated in monocytes [9,10], emphasize the requirement for divergent pathways and selective regulation of their activation. One cellular system that relies on sequential but distinct cellular responses to BMPs is the development of sensory projection neurons in the dorsal horn of the spinal cord. BMPs supplied by the roof plate initially specify the fates of several subsets of dorsal interneurons (dI neurons), directing expression of dI neuron class-specific transcription factors [11-14]. Subsequently, BMPs orient the axons of these post-mitotic dI neurons, directing their growth away from the dorsal midline [3,4,15] and also regulate the rate of growth of dI axons as they extend through the spinal cord [16]. Both orientation and rate of growth appear to occur within minutes *in vitro*, suggesting they are regulated independently of the early inductive BMP pathways. Moreover, intriguingly, whereas the two highly related roof plate-derived BMPs, BMP7 and BMP6, both induce the differentiation of dI neurons [3,4,12,13], BMP7, but not BMP6, is also able to orient dI axons *in vitro *and is required for appropriate dI axon projections *in vivo *[3,4].

How BMPs signal the distinct activities in spinal neurons is unclear. The slow time course and molecular changes in dI neuronal specification in response to BMPs imply activation of a nuclear signaling pathway. The core pathway underlying the transduction of BMP signals from the surface of a cell to the nucleus typically involves ligand-induced recruitment and activation of a BMP receptor complex, which comprises one pair each of type I and type II receptor subunits. BMP binding promotes phosphorylation of type I by type II BMP receptors [17,18]. Activated type I BMP receptors phosphorylate receptor-associated Smad1/5/8 proteins, resulting in nuclear translocation of Smad complexes and activation or repression of transcription of BMP target genes [18,19]. In monocytes, BMP7 and BMP6 activate Smad1/5/8 phosphorylation and Smads are required for gene induction [10]. However, a role for Smads as intracellular mediators in the induction of dI neuron-specific genes by BMPs has not been demonstrated and the question of how this pathway is transduced remains unsolved. In contrast to BMP-induced neural specification, the rapid time course of BMP-evoked growth cone orienting responses of dI neurons points to the recruitment of acute, transcription-independent pathways [3]. Although there is a growing appreciation of the existence of transcription-independent responses to BMPs, much less is known about acute BMP signaling than its classical inductive counterpart. In monocytes, Smad4 appears not to be required for BMP7-evoked chemotaxis [10]. Moreover, although in monocytes and other cell systems, effectors of cytoskeletal dynamics, such as PI3K, LIMK, and Rho family GTPases have been implicated as mediators of BMP-stimulated responses [10,16,20,21], their role in BMP-evoked axon orientation in dI neurons remains to be determined. Indeed, recent studies suggest that the activation of LIMK by BMPs regulates the rate of extension of dI axons, but not their orienting response to BMP7 [16].

Elucidating signaling components is an important step towards understanding the differential selection of transduction pathways, but how might BMPs activate distinct intracellular signaling pathways? Experiments on BMP7-evoked gene induction and chemotaxis in monocytic cells suggest that recruitment of different canonical BMP receptor subunits may represent an early step in triggering divergent signaling paths. Most tellingly, although it seems likely that type II BMP receptors are required, the inductive pathway does not appear to depend on a specific type II receptor, whereas the selective involvement of two of the three known type II BMP receptor subunits, ActRIIA and BMPRII, is required for BMP7-evoked chemotaxis [10]. The view that activation of particular type II BMP receptors is sufficient to initiate transcription-independent, acute cellular responses is supported by the observation that PI3K and LIMK can bind directly to the intracellular domains of type II BMP receptors [22-24]. Moreover, the BMPRII subunit has been implicated in eliciting LIMK-dependent responses to BMPs [16,22]. However, the evidence that type II BMP receptors direct acute signaling that diverges from the classical inductive events does not resolve whether they act in the context of the canonical type I/type II BMP receptor complex. Type I BMP receptor activity has been linked previously with activation of transcriptional BMP responses [14,25,26]. Nevertheless, the loss of BMPRIB in dI neurons and in ventral retinal ganglion neurons results in aberrant axon guidance [27,28]. From all these studies, a model is emerging in which canonical type I and type II BMP receptors support both the inductive specification and axon orienting activities of BMPs but the nature of the complex that drives orientation and the role of the individual receptor subunit activity remain unclear.

In the light of these findings, we have begun to resolve how BMPs exert their dual developmental effects on dI neurons by further evaluating the contributions of BMP receptor subunits and downstream signaling pathways to the inductive specification and axon orienting activities of BMP7. We have also examined how the selectivity of such responses is achieved. We have exploited the difference in axon orienting ability between BMP7 and BMP6, comparing requirements for their activities in neurons isolated in dissociated culture and in spinal explants. We demonstrate divergent BMP signaling pathways that operate concomitantly: a classical type I BMP receptor kinase-mediated path to BMP7-evoked Smad activation and neural specification, and a pathway dependent on PI3K activity, which independently mediates the orienting response of spinal axons to BMP7. Our results suggest a model in which BMP-evoked inductive specification in the dorsal spinal cord depends on type I BMP receptor activity and involves classical Smad signaling to the nucleus, whereas BMP7-elicited axon orientation depends on activation of PI3K signaling independent of type I BMP receptor activity and the Smad cascade, through differential engagement of type II BMP receptor subunits.

## Results

### Different concentration thresholds for Smad activation and growth cone collapse

**Figure 1:**
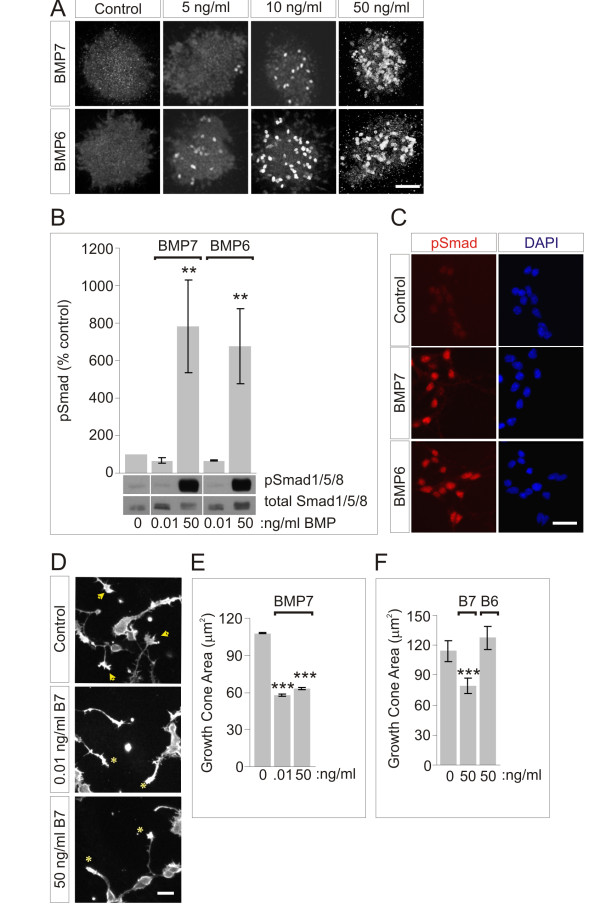
**dI induction, Smad activation and growth cone collapse by BMPs**. **(A) **Lhx2/9 induction in [i] explants treated with BMP7 or BMP6. Scale = 50 μm. **(B) **pSmad1/5/8 response to BMP7 or BMP6 in dI neurons. Western blots of whole cell lysates. Results (mean ± SEM), normalized to total cellular Smad1/5/8, are expressed as percent control pSmad1/5/8 (at 0.01 ng/ml BMP7 = 68 ± 13% and BMP6 = 66 ± 4% (n = 2); at 50 ng/ml BMP7 = 780 ± 247% and BMP6 = 675 ± 199% (n = 5)). Mann-Whitney tests: the pSmad1/5/8 response at 50 ng/ml BMP7 and BMP6 was significantly different from control (**P < 0.01); the pSmad1/5/8 response at 0.01 ng/ml BMP7 and BMP6 was not different from control (*P *= 0.0952). **(C) **Dissociated dI neurons ±50 ng/ml BMP7 or BMP6, double-labeled with phospho-specific α-Smad1/5/8 (red) and DAPI (blue): 95.2 ± 2.0% (BMP7) and 97.9 ± 1.5% (BMP6) dI neurons were pSmad1/5/8^+^. Scale = 20 μm. **(D) **ERM)-labeled dissociated dI neurons ±BMP7 at 0.01 or 50 ng/ml. Arrowheads indicate typical widespread growth cones and asterisks indicate collapsed growth cones. Scale = 20 μm. **(E) **Average growth cone areas (mean ± SEM): control = 107.6 ± 0.44 μm^2^; BMP7 (0.01 ng/ml) = 57.8 ± 1.11 μm^2^; BMP7 (50 ng/ml) = 63.4 ± 0.85 μm^2^. Areas measured in 0.01 or 50 ng/ml BMP7 differ significantly from control (***P < 0.001, Student's *t*-test). Results are for 140 to 380 growth cones/condition/experiment; n = 2. **(F) **Comparison of dI growth cone responses to BMP7 and BMP6 (50 ng/ml), measured as in (E): control = 114.3 ± 10.7 μm^2^; BMP7 = 79.5 ± 7.4 μm^2^; BMP6 = 127.4 ± 11.9 μm^2^. Student's *t*-tests: area in BMP7 differs significantly from control (***P < 0.001); area in response to BMP6 was not different from control (*P *= 0.0607). Results are for 100 to 115 growth cones/condition/experiment; n = 2.

We assessed whether there are differences in the initiation of BMP-evoked events in dI neurons, examining whether the inductive specification and axon orienting actions of BMP7 on dI neurons are evoked at different ligand concentrations. Initially, to determine an effective concentration range, we monitored the threshold for induction of dI1 neurons, a major class of spinal projection neurons. Explants of chick intermediate neural tube ([i] explants) were exposed to a range of BMP concentrations and examined after 48 h for the differentiation of dI1 neurons, marked by expression of the LIM homeodomain proteins Lhx2 and Lhx9 [11,29]. The threshold for expression of dI1 neuronal markers was approximately 5 ng/ml BMP7 or BMP6 (Figure 1A), with robust Lhx2/9 expression observed at 50 ng/ml (Figure 1A).

We now examined whether different classes of BMP response can be evoked concomitantly in individual dI neurons and whether these responses are initiated at different BMP ligand concentrations. We monitored BMP-evoked phosphorylation of Smad1/5/8 as an early step in the classical transcriptional signaling pathway. Smad1/5/8 phosphorylation was measured both by western blot analysis of dI neuronal lysates and by immunofluorescent labeling of dI neuron cultures. In sister cultures, we also measured growth cone collapse, as an example of an acute response to BMP7, occurring within minutes, and considered a surrogate for the axonal orientation response [3]. Growth cone collapse in the presence of BMPs was compared by measuring the growth cone area in dI neuron cultures, using ezrin-radixin-moeisin (ERM) immunoreactivity to visualize the growth cone [3].

Cultures of dissociated dI neurons were exposed to BMP7 and BMP6 at two concentrations: 50 ng/ml, based on the observation of dI neuronal specification in [i] explants (Figure 1A), and 0.01 ng/ml, a concentration sufficient to elicit monocyte chemotaxis [10]. At 0.01 ng/ml neither BMP7 nor BMP6 evoked Smad1/5/8 phosphorylation (Figure 1B), but at 50 ng/ml both ligands stimulated phosphorylation of Smad1/5/8 (7.8- and 6.8-fold over control values for BMP7 and BMP6, respectively; Figure 1B), with phospho-Smad1/5/8 (pSmad1/5/8) labeling detected in > 95% of all neurons (Figure 1C). In sister cultures, BMP7 elicited similarly robust growth cone collapse at both test concentrations, causing 46% (0.01 ng/ml) and 41% (50 ng/ml) decreases in the average growth cone area of dI neurons (Figure 1D,E). In contrast, BMP6 did not elicit growth cone collapse (Figure 1F). Although technical difficulties prevent the use of both ERM and pSmad1/5/8 immunoreactivity in the same cells, in sister cultures 50% of neurons showed growth cone collapse and 95% showed Smad1/5/8 phosphorylation. These results show that BMP7 stimulates both pSmad1/5/8 activation and growth cone collapse in individual neurons, that BMP6 can elicit only pSmad1/5/8 activation, and that these activities are elicited at different threshold concentrations of BMP7.

### Type I BMP receptor signaling participates in inductive specification but not axon orientation

Distinct thresholds for BMP-evoked inductive specification and axonal orientation raise the possibility that different receptor proteins signal these two activities, supporting the findings suggesting differential roles for type I and type II receptors in spinal cord and in monocytes [10,28]. We therefore explored whether the inductive and orienting responses of spinal neurons to BMP7 involve the activity of different BMP receptor subunits and/or intracellular signaling pathways. Type I BMP receptors are classically associated with activation of the Smad cascade. However, knock-down experiments have implicated the type I BMP receptor BMPRIB in roof plate-evoked spinal axon orientation [28]. We now further examined the roles of type I BMP receptors in BMP-evoked Smad activation and dI neuron inductive specification and in axon orientation by testing the consequences of blocking the activity of type I BMP receptor kinase. We used dorsomorphin (DM), an inhibitor of type I BMP receptor kinase activity [30,31], to assess the requirement for the activity of type I BMP receptors in dissociated dI neurons.

**Figure 2:**
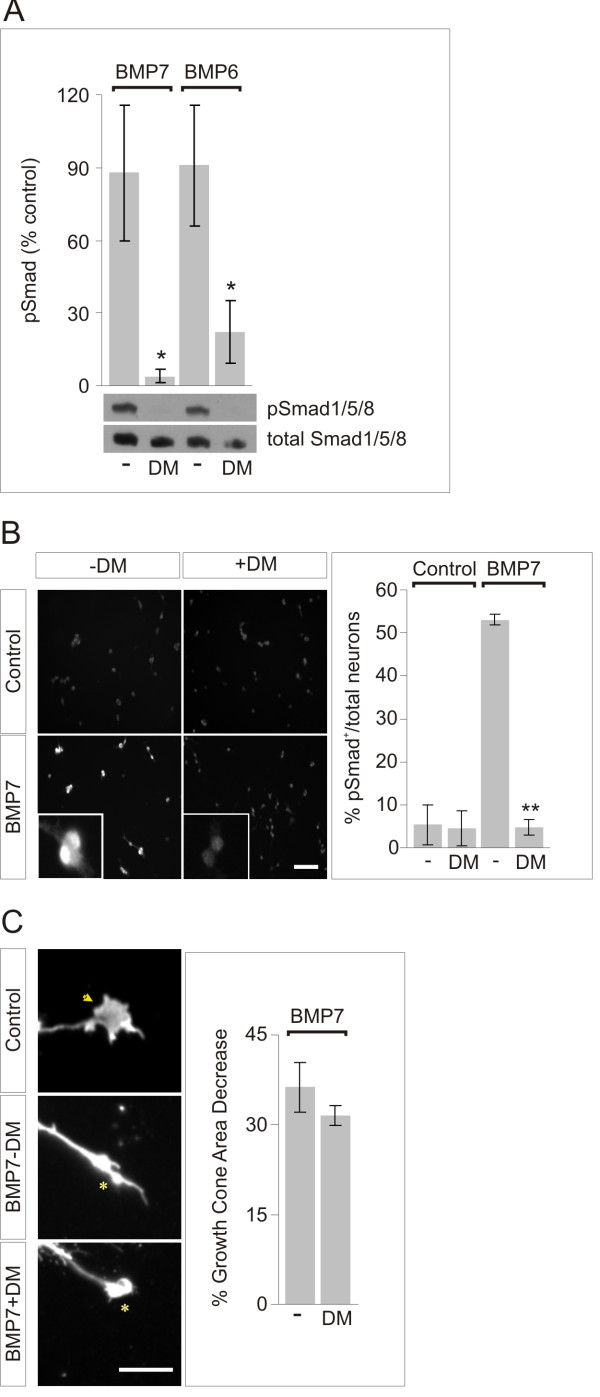
**BMP-evoked Smad1/5/8 phosphorylation, but not collapse activity, depends on type I BMP receptor kinase activity**. **(A) **Whole cell lysates of dissociated dI neurons with or without DM (10 μM) and with or without BMP7 or BMP6 (50 ng/ml), probed and normalized on western blots as in Figure 1B. Results are expressed as percentage of control pSmad1/5/8 for each condition (BMP7 - DM = 188 ± 28%; BMP7 + DM = 104 ± 3%; BMP6 - DM = 191 ± 25%; BMP6 + DM = 122 ± 13%; n = 2). DM had no effect on baseline pSmad1/5/8 (control + DM = 100 ± 13%). pSmad1/5/8 responses to BMP7 and BMP6 treated with DM differ significantly from pSmad1/5/8 responses to BMP7 and BMP6 without DM (*P < 0.05, Mann-Whitney test). **(B) **Left: pSmad1/5/8 in dissociated dI neurons with or without BMP7 and with or without DM. Scale = 80 μm. Insets: pSmad1/5/8-positive and -negative neurons (scale = 10 μm). Right: the percentage of pSmad1/5/8^+ ^neurons in control cultures with or without DM was unchanged (control - DM = 5.4 ± 4.7%; control + DM = 4.5 ± 4.1%; *P *= 0.8977, Student's *t*-test). The number of pSmad1/5/8^+ ^cells in response to BMP7 with or without DM differed significantly (BMP7 - DM = 53.1 ± 1.2%; BMP7 + DM = 4.7 ± 1.8%; **P < 0.005, Student's *t*-test). Results are mean ± SEM for four 20 × fields: 150 to 500 neurons/condition/experiment (n = 2). **(C) **ERM-labeled dissociated dI neuron sister cultures, with or without BMP7 and with or without DM as in (B). The growth cone area in control cultures was not changed by DM (not shown; control - DM versus control + DM, *P *= 0.9215 (Student's *t*-test)). The percentage decrease in growth cone area in response to BMP7 was not significantly altered by DM: control + DM = 1.3 ± 11%; BMP7 - DM = 36 ± 4%; BMP7 + DM = 32 ± 2%; n = 2; BMP7 - DM versus BMP7 + DM, *P *= 0.4097 (Student's *t*-test)). Arrowhead indicates control growth cone and asterisks indicate collapsed growth cones. Scale = 10 μm.

We first examined the effect of DM on levels of Smad1/5/8 phosphorylation evoked by 50 ng/ml BMP7 or BMP6. Initially, we tested a range of DM concentrations to determine an effective dose (see Materials and methods). At 10 μM, DM eliminated BMP-induced Smad1/5/8 phosphorylation, measured by both western blot analysis of whole cell lysates (Figure 2A) and immunofluorescent pSmad1/5/8 labeling in intact neurons (Figure 2B), indicating blockade of type I BMP receptor activity. We next assessed whether BMP7-evoked growth cone collapse was affected by DM in sister cultures of dissociated dI neurons. Exposure to BMP7 (50 ng/ml) evoked a 36% decrease in the average growth cone area of dI neurons (Figure 2C). DM (10 μM) had no significant impact on the growth cone collapsing activity of BMP7 (BMP7 + DM = 32% decrease in growth cone area; Figure 2C). Thus, DM effectively inhibits BMP-evoked Smad1/5/8 phosphorylation but not growth cone collapse in dI neurons. These data provide evidence that type I BMP receptor kinase activity is not required for BMP7-evoked growth cone collapse. They also indicate that activation of cytoskeletal dynamics by BMP7 occurs through a pathway distinct from the Smad cascade.

**Figure 3:**
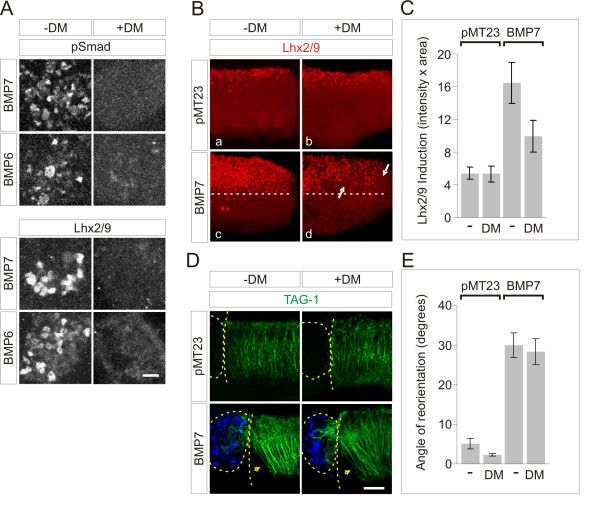
**Type I BMP receptor kinase activity required for BMP7-evoked Lhx2/9 induction, but not axon orientation**. **(A) **[i] explants treated with BMP7 or BMP6 (20 ng/ml) with or without DM (5 μM): α-pSmad1/5/8 (upper) and α-Lhx2/9 (lower) panels. Scale = 20 μm. **(B-E) **[d] explants, with appended control or BMP7-expressing COS-1 cells, with or without 10 μM DM. Explants co-labeled with α-Lhx2/9 (red) (B), and α-TAG-1 (green) and α-flag (blue) (C). Scale = 50 μm. (**B**) Endogenous Lhx2/9, induced prior to explantation, present only in the dorsal-most region of explants, cultured alone (not shown) or with pMT23-expressing COS-1 cells (a). DM did not alter endogenous Lhx2/9 (b). Dashed lines indicate ectopic ventral Lhx2/9 expression in [d] explants + BMP7-expressing COS-1 cells (c). DM treatment greatly reduced BMP7-induced ectopic Lhx2/9 (d, arrows). (**C**) Integrated density of Lhx2/9 expression (mean × 10^5 ^± SEM for each condition): pMT23 - DM = 5.41 ± 0.77 (n = 3); pMT23 + DM = 5.33 ± 0.94 (n = 2); BMP7 - DM = 16.46 ± 2.51 (n = 5); BMP7 + DM = 9.94 ± 1.90 (n = 6). (**D**) Dashed lines mark appended borders of COS-1 cell aggregates and [d] explants. Arrowheads indicate dI axons repelled by BMP7. (**E**) Angles of reorientation in [d] explants with pMT23- or BMP7-expressing COS-1 cells with or without DM (pMT23 = 5.1 ± 1.3° (n = 3); pMT23 + DM = 2.3 ± 0.3° (n = 2); BMP7 = 30 ± 3.1° (n = 5); BMP7 + DM = 28.4 ± 3.3° (n = 5). Results are mean ± SEM for each condition. Student's *t*-tests: DM had no effect on the D-V projection of dI axons either in control (pMT23 versus pMT23 + DM; *P *= 0.1936) or BMP7-orienting conditions (BMP7 versus BMP7 + DM; *P *= 0.7345).

We next examined the impact of type I BMP receptor kinase blockade on the specification and axonal orientation of dI neurons within spinal cord explants. In [i] explants, analysis of BMP-evoked stimulation of pSmad1/5/8 confirmed that phosphorylation of Smad1/5/8 by both BMP7 and BMP6 is abolished by treatment with DM (Figure 3A, upper panels). The ability of DM to alter BMP-evoked induction of Lhx2/9^+ ^cells was tested in [i] explants, in which individual cells expressing Lhx2/9 can be counted. In control explants, BMP7 induced expression of Lhx2/9 (7.7 ± 1.0 Lhx2/9^+ ^cells per 100 μm^2^; Figure 3A, lower panels). Treatment of [i] explants with BMP6 yielded similar results (8.6 ± 1.6 Lhx2/9^+ ^cells per 100 μm^2^; Figure 3A, lower panels). In the presence of DM (5 μM), induction of Lhx2/9 by both BMP7 and BMP6 was abolished (0 cells; Figure 3A, lower panels). Thus, DM blocks Smad1/5/8 phosphorylation and dI1 neuronal specification by BMPs in spinal [i] explants.

Based on these findings, we monitored the effects of DM in explants of rat dorsal spinal cord ([d] explants), in which BMP-evoked Lhx2/9 induction and dI axon orientation can be examined in parallel [3,4]. In control explants cultured adjacent to pellets of COS-1 cells expressing an empty vector, expression of Lhx2/9 was restricted to dorsal regions of the explants (Figure 3B(a)) with a pattern similar to that observed in sections of embryos taken at the same age (not shown). Endogenous Lhx2/9 expression was unaffected by DM treatment (Figure 3B(b)). In [d] explants co-cultured with BMP7-expressing COS-1 cells, ectopic Lhx2/9 expression was observed (Figure 3B(c)). The dorsal to ventral (D-V) extent of Lhx2/9 expression was expanded, reflecting a 3.1-fold increase in dI1 neurons (Figure 3B(c),3C). DM treatment (10 μM) substantially reduced ectopic Lhx2/9 expression induced by BMP7 in [d] explants by 39% (BMP7 + DM = 1.9-fold over control; Figure 3B(d),3C), corroborating the result in [i] explants (Figure 3A) and providing further evidence that BMP-mediated specification of dI1 neurons require type I BMP receptor kinase activity. The effect of DM on the orientation of TAG-1^+ ^dI axons was examined in the same [d] explants as those used to measure Lhx2/9 induction (Figure 3B). In [d] explants cultured adjacent to control COS-1 cells expressing empty vector, dI axons extended with a D-V trajectory (angle of reorientation (pMT23) of 5.1 ± 1.3°; Figure 3D,E). In [d] explants exposed to BMP7-transfected COS-1 cells, TAG-1^+ ^axons were repelled, extending away from the BMP7 source, with an angle of reorientation of 30 ± 3.1° (Figure 3D,E). Although ectopic Lhx2/9 expression was reduced when DM (10 μM) was applied to the [d] explants (Figure 3B,C), no change was observed in the response of dI axons to BMP7 in the presence of DM (angle of reorientation of 28.4 ± 3.3°; Figure 3D,E). The lack of effect of DM on dI axon orientation parallels the resistance of BMP7-evoked growth cone collapse to blockade of type I BMP receptor kinase activity (Figure 2C).

From these results we infer that type I BMP receptor activity, potentially acting through the Smad cascade, initiates the BMP7- and BMP6-evoked pathway of inductive specification, but although type I receptor subunits may be required as part of the functional receptor complex [28], type I BMP receptor kinase activity is not required for BMP7-evoked axon orientation. Taken together, our results argue that divergence of dI neuron inductive and orienting responses stems from distinct BMP:receptor interactions in which BMP7, at low concentrations, and BMP7 and BMP6, at high concentrations, engage different receptors within the receptor complex. These considerations led us to examine which type II BMP receptors and associated downstream signaling components might support axon orientation selectively.

### dI neurons express type II BMP receptors

**Figure 4:**
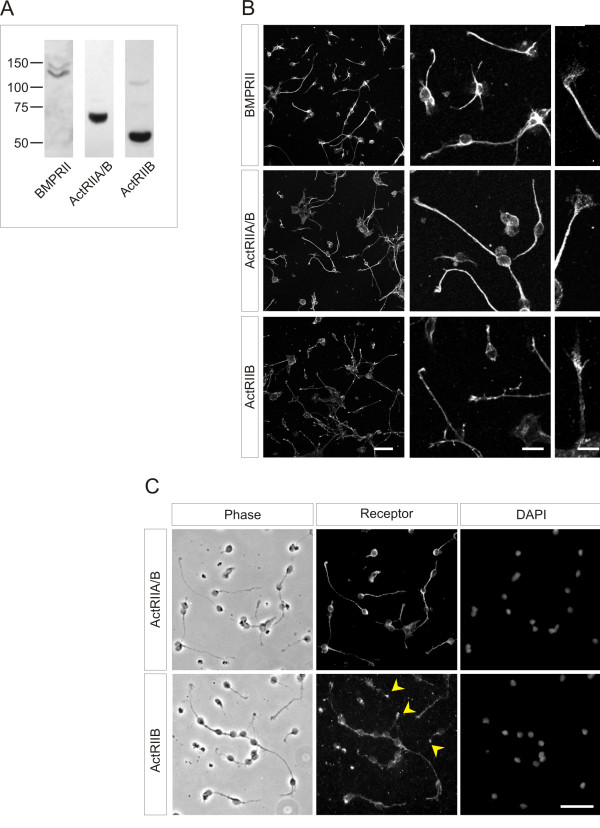
**Type II BMP receptor expression in dI neurons**. **(A) **Western analysis of whole cell lysates of dissociated dI neurons probed with α-BMPRII (130 kDa, lane 1), α-ActRIIA/B (70 kDa, lane 2) and α-ActRIIB (58 kDa, lane 3) antibodies. **(B) **Immunofluorescence labeling of dissociated dI neurons with antibodies against type II BMP receptors (α-BMPRII, α-ActRIIA/B and α-ActRIIB). The left column shows relative numbers of labeled neurons against background low level fluorescence (scale = 50 μm). The center column (scale = 20 μm) and right column (scale = 10 μm) highlight neuronal detail. **(C) **Direct comparison of ActRIIA and ActRIIB expression in sister cultures. Phase contrast (left), BMP receptor immunofluorescence labeling (center) and DAPI nuclear staining (right) of dissociated dI neurons. Arrowheads indicate ActRIIB^+ ^growth cones. Analysis of immunofluorescence label for each antibody from three to four 20 × fields showed that 85.6 ± 3.7% of 1,056 total neurons were ActRIIA^+ ^(n = 4), 33.1 ± 3.7% of 732 total neurons were ActRIIB^+ ^(n = 4) and 78.6 ± 3.3% of 465 total neurons were BMPRII^+ ^(n = 3). Scale = 50 μm.

Of the three type II BMP receptors, only ActRIIA and BMPRII are required for Smad-independent BMP7-evoked chemotaxis of monocytic cells [10]. Nothing is known in detail of the *in vivo *distribution of type II BMP receptors in embryonic spinal cord. To begin to explore the possibility that BMP7-evoked growth cone collapse and orientation involves specific type II BMP receptors, we determined the distribution of all three type II BMP receptors in dissociated dI neurons. Western blots of dI neuronal lysates showed expression of all three type II BMP receptors (Figure 4A). Immunofluorescence analysis of dI cultures, in conjunction with phase contrast imaging and DAPI nuclear staining to provide total neuronal counts, revealed that ActRIIA and BMPRII are the predominant type II BMP receptors expressed in dI neurons, in 86% (ActRIIA) and 79% (BMPRII) of neurons, respectively (Figure 4B,C). In contrast, ActRIIB was expressed in only 33% of neurons. ActRIIA and BMPRII are expressed by processes, growth cones and cell bodies (Figure 4B,C), whereas ActRIIB is expressed mainly in growth cones (Figure 4B,C). Thus, the type II receptors necessary for BMP7-evoked chemotaxis, ActRIIA and BMPRII, are prominently expressed by dI neurons.

### BMP7-mediated dI inductive specification is independent of PI3K signaling

The observations described in the previous section prompted us to consider the roles of signaling pathways associated with type II BMP receptors in BMP7-evoked neuronal activities. We explored the possibility that a pathway mediated by PI3K might elicit axon orientation independently of inductive specification. PI3K and LIMK1 are both associated with type II BMP receptors [22,23]. Moreover, cell migration and chemotaxis of non-neuronal cells in response to BMP2 [21,32] and BMP7 [10] are dependent on PI3K, whereas LIMK appears to regulate rate, but not direction of, dI axon extension within the spinal cord [16]. We therefore examined the role of PI3K activity in BMP-evoked inductive specification and axon orientation in spinal explants, using the inhibitors of PI3K activity, LY294002 (LY) and wortmannin (WM) [33].

**Figure 5:**
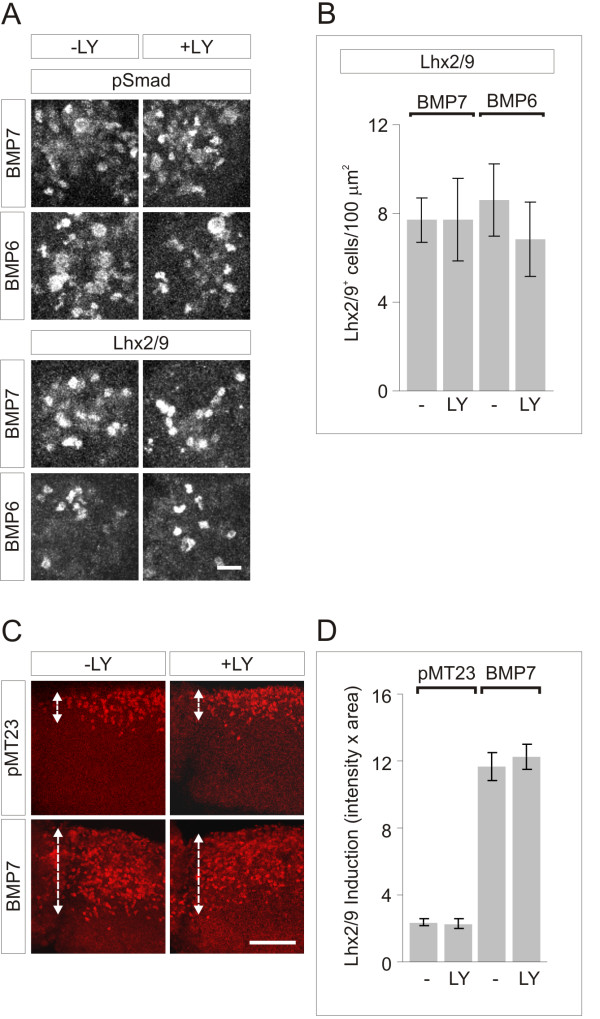
**Inhibition of PI3K activity has no effect on BMP7-induced Lhx2/9 expression**. **(A) **[i] explants treated with BMP7 or BMP6 (20 ng/ml) with or without 2.5 μM LY and labeled with α-pSmad1/5/8 (upper panels) and α-Lhx2/9 (lower panels). Scale = 20 μm. **(B) **Quantification of Lhx2/9^+ ^cells/100 μm ^2 ^in [i] explants: BMP7 - LY = 7.7 ± 1.0 cells; BMP7 + LY = 7.7 ± 1.9 cells; BMP6 - LY = 8.6 ± 1.6 cells; BMP6 + LY = 6.8 ± 1.7 cells; (n = 2) (mean ± SEM for each condition). LY had no effect on Lhx2/9 expression induced by either BMP7 or BMP6 (BMP7 - LY versus BMP7 + LY, *P *= 0.9997; BMP6 - LY versus BMP6 + LY, *P *= 0.4942 (Student's *t*-test)). **(C) **Lhx2/9-labeled (red) [d] explants co-cultured with control- or BMP-7-expressing COS-1 cells with or without 2.5 μM LY. Double-headed arrows mark the width of the control and induced Lhx2/9-expression regions. Scale = 50 μm. **(D) **Quantification of Lhx2/9 induction in [d] explants, measured as in Figure 3C: pMT23 - LY = 4.48 ± 0.44 (n = 8); pMT23 + LY = 4.85 ± 0.55 (n = 8); BMP7 - LY = 14.02 ± 1.0 (n = 17); BMP7 + LY = 12.42 ± 0.78 (n = 18). Mean × 10^5 ^± SEM for each condition. Student's *t*-tests: there was no difference in the expression levels of Lhx2/9 with or without LY in either control [d] explant co-cultures (pMT23 - LY versus pMT23 + LY, *P *= 0.8002) or BMP7-induced [d] explants (BMP7 - LY versus BMP7 + LY, *P *= 0.5978).

As shown above (Figure 3A), BMP7 and BMP6 stimulated phosphorylation of Smad1/5/8 and induction of Lhx2/9 in [i] explants (Lhx2/9^+ ^cells: BMP7 = 7.7 ± 1.0 cells; BMP6 = 8.6 ± 1.6 cells; Figure 5A,B). Incubation of [i] explants with LY (2.5 μM) had no effect on BMP7-evoked pSmad1/5/8 or Lhx2/9 induction (Lhx2/9^+ ^cells: BMP7 + LY = 7.7 ± 1.9 cells; BMP6 + LY = 6.8 ± 1.7 cells; Figure 5A,B). Similarly, in [d] explants, LY treatment had no effect on the inductive response to BMP7: co-culture of BMP7-expressing COS-1 cells with [d] explants induced ectopic Lhx2/9 expression (4.9-fold over control levels; Figure 5C,D). In the presence of LY (2.5 μM), [d] explants exposed to BMP7 showed a 5.3-fold increase in Lhx2/9 expression that was not significantly different from [d] explants without LY (Figure 5C,D). Together, these results provide evidence that neither the phosphorylation of Smad1/5/8 nor the intracellular events underlying neural specification by BMP7 employ PI3K as a signaling intermediate.

### PI3K involvement in BMP7-mediated growth cone collapse and axon orientation

**Figure 6:**
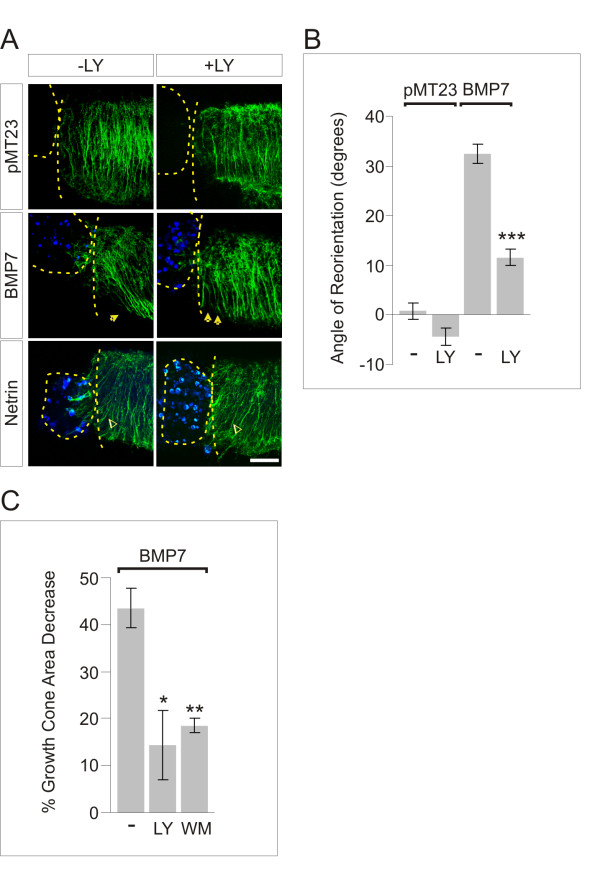
**BMP7-evoked growth cone collapse and axon orientation is dependent on PI3K activity**. **(A) **[d] explants cultured as in Figure 5C with control- or BMP7- (blue; α-flag) or Netrin-1- (blue; α-Netrin) expressing COS-1 cells, with or without 2.5 μM LY (+LY). Green shows TAG-1-labeled axons. Dashed lines mark COS-1 cell aggregate and [d] explant appended borders. The closed single arrowhead indicates dI axons repelled by BMP7. The double closed arrowheads indicate angle of axons with LY. Open arrowheads indicate dI axons attracted by Netrin-1. Scale = 50 μm. **(B) **Angle of reorientation in [d] explants co-cultured with or without BMP7 and with or without LY (mean ± SEM for each condition): pMT23 = 0.8 ± 1.7° (n = 9); pMT23 + LY = -4.3 ± 1.8° (n = 3); BMP7 = 32.5 ± 1.9° (n = 18); BMP7 + LY = 11.6 ± 1.7° (n = 29). Student's *t*-tests: the straight D-V projection of axons in control [d] explants was unchanged by LY (pMT23 - LY versus pMT23 + LY, *P *= 0.1412). BMP7-evoked reorientation was significantly inhibited by LY (BMP7 - LY versus BMP7 + LY, ***P < 0.0001). Attraction of dI axons by Netrin-1 was unchanged by LY (Netrin - LY = 51.5 ± 3.5° (n = 3); Netrin + LY = 49.0 ± 1.5° (n = 4); Netrin - LY versus Netrin + LY, *P *= 0.4621). **(C) **BMP7-evoked dI growth cone collapse measured with or without LY or WM. Percentage decrease of growth cone area relative to control cultures (mean ± SEM): BMP7 = 44 ± 4% (n = 3); BMP7 + LY = 14 ± 7% (n = 2); BMP7 + WM = 19 ± 2% (n = 3). BMP7-stimulated reduction in growth cone area was significantly different from cultures treated with BMP7 + LY or BMP7 + WM (BMP7 - LY versus BMP7 + LY, *P < 0.05; BMP7 - WM versus BMP7 + WM, **P < 0.005; (Student's *t*-test)). Results are for 200 to 400 growth cones/condition/experiment.

We next measured the effect of LY on BMP7-evoked axon orientation in the same [d] explants in which Lhx2/9 expression was monitored (Figure 5C). In control [d] explants co-cultured with adjacent pellets of COS-1 cells expressing empty vector, axons extended with a straight D-V trajectory with an angle of reorientation of 0.8 ± 1.7° (Figure 6A,B). In [d] explants adjacent to BMP7-expressing COS-1 cells, axons were repelled, extending away from the BMP source with an angle of reorientation of 32.5 ± 1.9° (Figure 6A,B). LY (2.5 μM) significantly inhibited the ability of BMP7 to orient dI axons: in the presence of LY the angle of reorientation in response to BMP7 was 11.6 ± 1.7° (Figure 6A,B), a reduction of orientation of more than 60%. To control for non-specific effects of LY on orienting responses of axons in [d] explants, we examined the effect of LY on Netrin-1-evoked orientation of dI axons [34]. Axon orientation towards Netrin-1 was unaffected in the presence of LY (Netrin - LY = 51.5 ± 3.5°; Netrin + LY = 49.0 ± 1.5°; *P *= 0.4621 (Student's *t*-test); Figure 6A), indicating selective susceptibility of BMP7-evoked dI axonal responses to inhibition of PI3K signaling. Thus, the ability of BMP7 to orient dI axons appears dependent on PI3K signaling.

Members of the MAPK family and cAMP have been identified as intermediates in Smad-independent signaling downstream of BMPs and/or associated with axonal guidance responses in other systems [17,35], raising the possibility that they also function in BMP7-activated dI axonal orientation. We tested inhibitors of MAPKs and modulators of cAMP activity for their ability to regulate BMP7-evoked dI axon orientation in [d] explants. The angle of BMP7-evoked axonal reorientation was unchanged by an inhibitor of PKA (KT5720), by an adenylate cyclase agonist (forskolin), by an Erk1/2 MAPK inhibitor (PD98059) or by a p38 MAPK inhibitor (SB203580) (Additional file 1). These results provide further support for the idea that PI3K, rather than MAPK activity or cAMP-dependent signaling, mediates the dI axon orienting response to BMP7.

To summarize, treatment with LY selectively blocked BMP7-evoked axon orientation in the same explants in which ectopic Lhx2/9 expression was unaffected, suggesting that PI3K activity is required for the action of BMP7 on dI axon orientation but not dI1 neuronal specification. To control further for non-specific actions of LY, we tested a second inhibitor of PI3K activity, WM, in parallel with LY. We assessed the ability of LY and WM to regulate BMP7-evoked growth cone collapse in dissociated dI neurons. BMP7 alone evoked a 44% decrease in the average growth cone area of dI neurons (Figure 6C). Incubation of neurons with BMP7 and either LY (50 μM) or WM (100 nM) resulted in substantial (75% (LY) and 57% (WM)) reductions in growth cone collapse (BMP7 + LY = 14% decrease in average growth cone area; BMP7 + WM = 19% decrease in average growth cone area; Figure 6C). These results provide pharmacological evidence that BMP7-evoked dI growth cone collapse is mediated by a PI3K-dependent mechanism.

### BMP7, but not BMP6, activates PI3K-dependent downstream signaling

**Figure 7:**
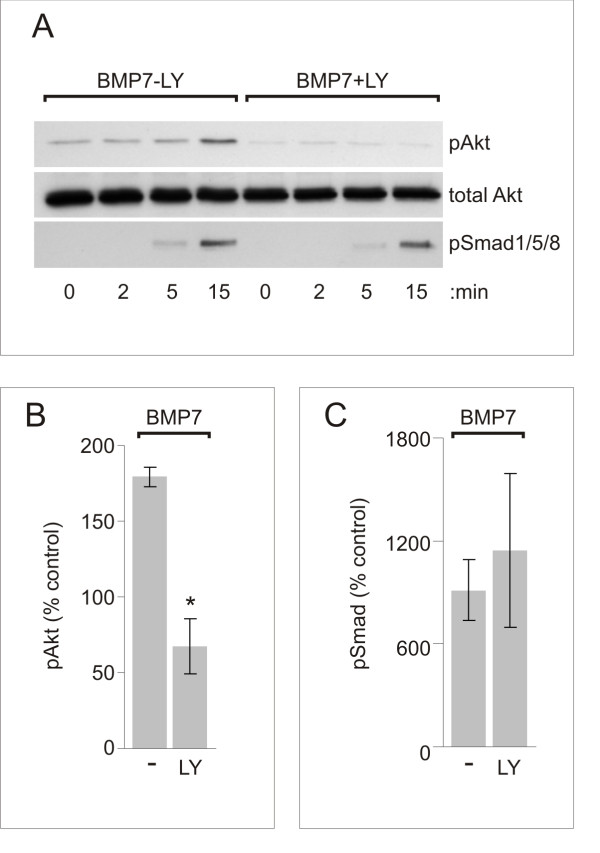
**BMP7 evokes rapid PI3K-dependent stimulation of Akt phosphorylation in dI neurons**. **(A) **Dissociated dI neurons pretreated with or without 50 μM LY and incubated with 50 ng/ml BMP7 for 0, 2, 5 or 15 minutes. Western blots of whole cell lysates probed with phospho-specific α-Akt or α-Smad1/5/8, then α-total cellular Akt protein (to normalize the pAkt signals). **(B, C) **pAkt (B) and pSmad1/5/8 (C) levels in the same dI neurons in response to 50 ng/ml BMP7 with or without LY, at 15 minutes. Percentage of control for each condition (mean ± SEM)): for pAkt, BMP7 - LY = 179 ± 6% and BMP7 + LY = 67 ± 18% (n = 2); for pSmad1/5/8, BMP7 - LY = 913 ± 175% and BMP7 + LY = 1,143 ± 448% (n = 2). Student's *t*-tests: BMP7-evoked pAkt in dI neurons with LY differs significantly from pAkt without LY (BMP7 - LY versus BMP7 + LY, *P < 0.05). BMP7-induced pSmad1/5/8 levels did not differ in dI neurons with or without LY (BMP7 - LY versus BMP7 + LY, *P *= 0.6798).

We next asked whether BMP7 can activate a PI3K-dependent pathway in dI neurons independent of the BMP7-evoked Smad and inductive specification pathway(s). As an indicator of PI3K activity, we used the LY-sensitive phosphorylation of a major downstream target of PI3K signaling, Akt [21,36]. The activity of BMP7 was tested at a concentration that evokes both induction and orientation. Dissociated dI neuron cultures were treated with 50 ng/ml BMP7 and whole cell lysates were collected over a series of time points and analyzed by western blot. After 15 minutes of BMP7 treatment, pAkt levels were substantially increased (pAkt level = 1.8-fold over control; Figure 7A,B) as was Smad1/5/8 phosphorylation (pSmad level = 9.1-fold over control; Figure 7A,C). Pretreatment with LY (50 μM) significantly reduced the increase in pAkt levels stimulated by BMP7 (BMP7 + LY pAkt level = 0.7-fold of control; Figure 7A,B). As in [i] explants, LY had no effect on BMP7-stimulated pSmad1/5/8 levels in cultures of dI neurons (BMP7 + LY pSmad level = 11.4-fold over control; Figure 7A,C). Thus, BMP7 appears to stimulate PI3K activity and by a pathway that is independent of Smad activation in dI neurons.

**Figure 8:**
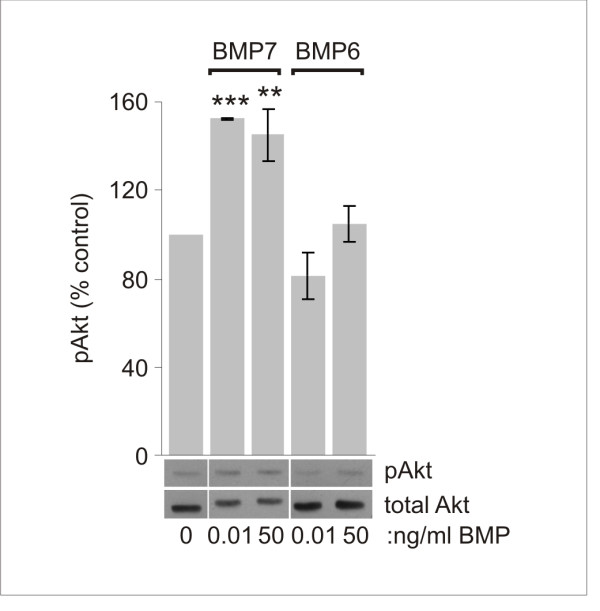
**BMP7 but not BMP6 stimulates Akt phosphorylation in dI neurons**. Whole cell lysates of dissociated dI neurons with or without BMP7 or BMP6, at 0.01 and 50 ng/ml for 30 minutes, probed on western blots with α-pAkt as in Figure 7A. Percentage of control for each condition relative to baseline pAkt levels (mean ± SEM): at 0.01 ng/ml, BMP7 = 153 ± 0.5%, and BMP6 = 82 ± 11% (n = 2); at 50 ng/ml, BMP7 = 145 ± 12%, and BMP6 = 105 ± 8% (n = 5). Student's *t*-tests: pAkt in dI neuron cultures treated with 0.01 or 50 ng/ml BMP7 was significantly different from control pAkt (**P < 0.01, ***P < 0.001). pAkt in cultures treated with 50 ng/ml BMP6 did not differ from control (*P *= 0.5628).

We next examined the selectivity of the Akt response to BMP7 by testing the two concentrations that distinguish BMP7 actions in neural induction and growth cone collapse (Figure 1). BMP7 stimulated the phosphorylation of Akt at both 0.01 and 50 ng/ml (1.53-fold and 1.45-fold over control, respectively; Figure 8). This result parallels the finding that BMP7 causes growth cone collapse at both concentrations, but distinguishes Akt activation from BMP7-stimulated Smad1/5/8 phosphorylation, which occurs only at high concentrations (Figure 1B). Thus, BMP7 stimulates PI3K activity at ligand concentrations consistent with a role for PI3K in the orienting response to BMP7. These observations led us to determine whether signaling through the PI3K-dependent mechanism is selectively activated by a BMP with orienting activity. We compared the abilities of BMP7 and BMP6 to phosphorylate Akt in dI neurons, using western blot analysis of dI neuron cultures treated for 15 minutes with 0.01 ng/ml or 50 ng/ml BMP7 or BMP6. As described above, BMP7 consistently evoked increases in pAkt (1.53- and 1.45-fold increases at the two concentrations). In contrast, BMP6 showed no increase in pAkt over levels in control cultures (0.8- and 1.1-fold of control, respectively; Figure 8). Taken together, these results provide evidence that BMP7 activates a PI3K-dependent pathway under conditions in which it stimulates the orienting response of dI neurons. Moreover, the ability of BMP7 to activate this path is selective to BMP7 over BMP6 and independent of Smad activation, suggesting that PI3K activity participates in a transduction pathway distinct from that mediating the inductive specification activity of BMPs.

## Discussion

**Figure 9:**
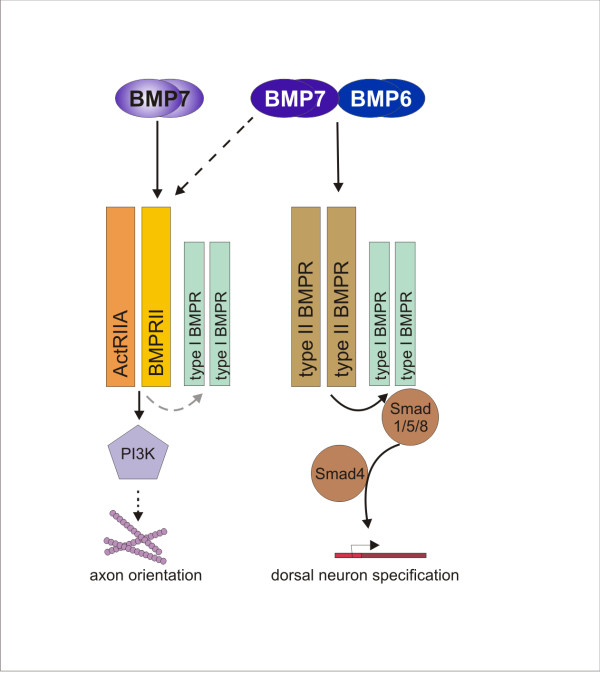
**Model of divergent BMP signaling pathways controlling transcriptional and cytoskeletal responses**. The distinct responses of dI neurons to BMP7 (neural induction and axon orientation) occur through independent pathways. The neural specification pathway, activated by both BMP7 and BMP6, at 'high' concentrations, leads to stimulation of the Smad cascade and induction of target genes. This signaling pathway requires the kinase activity of type I BMP receptors but appears not to depend on specific type II BMP receptors. The chemotropic response of dI neurons, axon orientation and growth cone collapse, is evoked by BMP7 but not BMP6, is activated at low concentrations of agonist and is not dependent on type I BMP receptor activity. Rather, this path involves PI3K-dependent signaling mechanisms and may be initiated through recruitment of a specific type II receptor pair selectively by BMP7.

We have examined the nature and divergence of signaling pathways that control transcriptional and cytoskeletal responses to BMP7 in dorsal spinal neurons. Intracellular BMP signaling is communicated through multiple pathways [17,37] and how and where those paths diverge or converge is still under study. One problem, illustrated here for dI neurons, is how a given BMP directs more than one kind of response in the same cell either concomitantly or sequentially. Our results cast light on this issue by demonstrating two pathways, one activated by both BMP7 and BMP6 and the other selectively by BMP7, which direct different cellular activities in dI neurons. These paths diverge upon receptor activation, suggesting a model (Figure 9) of recruitment of canonical BMP receptor subunits into distinct complexes. Under this paradigm, one consequence of BMP binding is dominated by type I BMP receptor activity leading to initiation of the Smad cascade and activation of nuclear responses. The second pathway recruits a receptor complex that leads to PI3K-dependent signaling, presumably to the cytoskeleton. Type II, rather than type I BMP receptors, are likely to play the major instructive role in this second pathway directing axon orienting responses. Although this model addresses the differential effects of BMP7 in dI neurons, the parallels with BMP-evoked events in monocytes suggest common principles underlying mechanisms of inductive and chemotropic BMP responses in a variety of cellular contexts.

### Divergent pathways activated concomitantly in single neurons

The two responses to BMP7 demonstrated in dI neurons are by their nature distinct. Although in dI neurons, *in vivo*, specification and axon guidance occur sequentially during development, in monocytes BMP7 activates chemotaxis and gene induction in the same cell concurrently. Similarly, in dissociated dI neurons we show here that the two divergent pathways can be activated concurrently. In experiments examining activity of BMP7 on individual neurons, sister cultures were always used. In dissociated dI neuron cultures, > 95% of cells show Smad1/5/8 phosphorylation in response to BMP7 and in sister cultures > 50% show growth cone collapse. Thus, in at least 45% of the neurons in culture both pathways are activated by the same dose of BMP7. The use of [d] spinal explants to demonstrate both induction and axon orientation in response to BMP7 does not assume that the same neurons responded in both ways but were used to compare the two responses under the same pharmacological manipulation.

### Distinct downstream pathways underlie inductive and acute BMP activities

Type I BMP receptor kinase activity appears to mediate the activation of the Smad pathway and inductive specification in dI neurons but is not involved in axonal orientation. The similar dose-dependent stimulation of Smad1/5/8 phosphorylation by BMP7 and BMP6 provides evidence that the Smad cascade is unlikely to mediate axonal orienting effects, which are selective to BMP7. Indeed, at concentrations at which BMP7 actively evokes growth cone collapse, the Smad pathway in dI neurons appears not to be engaged. In addition, in dissociated dI neurons and explants, BMP-evoked phosphorylation of Smad1/5/8 was blocked by DM whereas neither growth cone collapse nor axon orientation responses to BMP7 were affected. In contrast, ectopic dI1 neuronal differentiation marked by Lhx2/9 expression was blocked by inhibition of type I BMP receptor kinase activity with the associated blockade of Smad1/5/8 phosphorylation. Importantly, although Smad1/5/8 phosphorylation and dI neuronal specification respond similarly to all treatments, we have not established directly that Smad activity transduces BMP-evoked neural specification. Other activators downstream of type I BMP receptors may represent Smad1/5/8-independent mediators of inductive pathways signaled by BMPs [37,38]. Nonetheless, together, these findings indicate divergence of the signaling underlying the inductive and orienting responses of dI neurons to BMP7.

Type I BMP receptor kinase activity is not required for the chemotropic activity of BMP7, posing the question of how an axon orienting signal is generated. Three lines of evidence suggest that stimulation of PI3K activity represents a pathway selected by BMP7 to evoke axonal orientation and provide further support to the model for independence of BMP7 signaling pathways. First, at the low concentrations at which BMP7 stimulates growth cone collapse in dI neurons, PI3K-dependent signaling, but not Smad1/5/8 phosphorylation, is activated by BMP7. Second, BMP6 does not stimulate PI3K-dependent Akt activity in dI neurons at any concentration tested, paralleling its lack of orienting ability. Third, the blockade by LY and WM of BMP7-evoked growth cone collapse and orientation of spinal axons, but not of BMP-evoked neuronal specification, suggests the involvement of PI3K as a signaling component selective to the orienting activity of BMP7. Similar results were obtained with two distinct inhibitors, supporting the view that PI3K was the target and is a mediator in this pathway. PI3K is known to be an intermediate in pathways that regulate cell motility and migration [39,40], raising the possibility that indiscriminate blockade of growth cone cytoskeletal dynamics underlies the block of BMP7-evoked axon orientation. Against this argument, Netrin-1-evoked axon orientation was unaffected by blockade of PI3K activity, indicating selectivity of the BMP7-evoked pathway for axon orientation.

Several intracellular mediators, PI3K, LIMK and Rho GTPases, have been implicated in BMP-evoked chemotaxis [10,21], growth cone and axon orientation and the dynamics of axon extension ([16,20] and current study). How do these related signals conspire to elicit BMP-dependent cytoskeletal reorganization? Our results add to the increasing evidence for a BMP-evoked chemotropic signaling pathway that includes PI3K, likely activated by type II BMP receptors. Through the action of downstream pathway components, such as Rho GTPases, PI3K activation may lead to oriented regulation of cytoskeletal dynamics [40-43]. BMP-activated LIMK, in partnership with cofilin, may act in parallel to regulate the rate of response to the chemorepellent BMP7 [16]. Nonetheless, details of the interactions and hierarchies in this pathway remain to be determined.

### The origins of BMP7 signaling divergence

The problem of how morphogens elicit both inductive and tropic actions has recently been addressed for Wnt and Hh proteins. In these cases, regulation of intracellular responses appears to depend on differential expression and activation of canonical and distinct, non-canonical, receptors and co-receptors [5,6,44,45], although so far in separate cells. In a departure from this theme, BMPs appear to activate variously grouped subsets of a relatively small collection of canonical BMP receptors to elicit differential responses [10,18,28,46], which, importantly, can occur in an individual cell ([9,10] and this study). This suggests that the point of divergence of the cellular responses to the same BMP lies with differential canonical receptor engagement.

In this study we have not established how BMP7 engages receptors selectively. Smad1/5/8 phosphorylation and dI neuron specification occur only in response to relatively high doses of BMP7 or BMP6, suggesting a lack of receptor selectivity, a notion supported by observations on receptor redundancy in long-term BMP responses in monocytes and neurons [10,25,28,47]. In contrast, the inability of BMP6 to evoke chemotropic responses or activate downstream signaling relevant to cytoskeletal dynamics, at any of a wide range of concentrations tested, supports the idea of an orientation-specific receptor complex. The combination of type I and type II BMP receptor subunits that mediates orientation is activated by low concentrations of BMP7. This complex can also be activated at the substantially higher concentration at which BMP7 activates the inductive pathway, suggesting that BMP7 is able to recruit selectively the receptor complex involved in orientation.

Which receptor subunits comprise the complex that mediates orientation? We have demonstrated the requirement for an unusual pairing of type II BMP receptors, ActRIIA and BMPRII, in mediating the chemoattractant effects of BMP7 in monocytic cell chemotaxis [10]. Moreover, the type I BMP receptor BMPRIB has been implicated in dI axon guidance, with selectivity over BMPRIA [28]. Our finding that the kinase activity of type I BMP receptors is not necessary for BMP7-evoked growth cone collapsing activity or axonal orientation leads us to propose that the requirement for BMPRIB in axonal responses reflects a role for this subunit independent of its kinase activity, perhaps acting to maintain a particular structural conformation of the ActRIIA/BMPRII/BMPRIB receptor complex. Whether BMPRIB is required physically to support the functional activation of a discrete set of type II BMP receptor subunits that direct the PI3K-dependent cascade to axon orientation needs further studies.

How might a distinct assembly of type II BMP receptor subunits direct the orienting responses of chemotropic BMPs? The choice of downstream pathway may depend on the mode of receptor oligomerization upon binding to BMP7: whether BMPs bind to preformed receptor complexes present in the membrane or initiate ligand-induced receptor complex assembly has been shown to dictate cellular response [48,49]. Distinct signaling information generated by identical receptors arranged variably within the receptor complex [50,51] seems unlikely in the case of dI neurons since BMP7 continues to orient axons at the higher concentration required for the inductive response. In dI neurons, the thresholds for BMP7 activation of Smad1/5/8 and PI3K (and accompanying inductive specification and orienting responses) suggest that different affinities of BMPs for distinct receptor subunits influence signaling outcomes. Further studies will be required to understand how BMP7 achieves selective recruitment of BMP receptors and the details of how this translates into differential activity of two signaling pathways.

## Conclusions

In dorsal spinal neurons inductive and axon orienting responses represent sequential steps in the differentiation of single neurons. We show here, however, that the two classes of response can also be evoked concurrently in individual dI neurons. Our results suggest that inductive specification and axonal orientation arise from activation of different receptor complexes. The activation of Smads and associated specification of dI1 neurons by BMP7 or BMP6 depend on type I BMP receptor activity. Conversely, the ability of BMP7 to orient axons and growth cones does not depend on a pathway initiated by type I BMP receptor activity, relying instead on a distinct cascade of cytoskeletal activators, including PI3K, that likely result from engagement of type II BMP receptors.

## Materials and methods

### Antibodies and reagents

Recombinant BMPs were purchased from R&D Systems, Minneapolis, MN, USA, and stock solutions were prepared in 4 mM HCl/0.1% BSA. Pharmacological reagents: LY (Cell Signaling Technology, Danvers, MA, USA) and WM (Sigma (St Louis, MO, USA)) for PI3K; DM (Sigma) for type I BMP receptor activity; PD98059 for Erk1/2 MAPK (Calbiochem, San Diego, CA, USA), SB203580 for p38 MAPK (Calbiochem), KT5720 for protein kinase A inhibition (Calbiochem) and forskolin for adenylate cyclase activation (Calbiochem). Each stock solution for the pharmacological reagents was prepared in DMSO and subsequently diluted in medium as specified. Antibodies were: mouse α-TAG-1 (4D7 [52]); rabbit α-Lhx2/9 (L1 [11]); mouse α-ERM (13H9 [53]); rabbit α-Smad1/5/8 (N18; Santa Cruz Biotechnology, Santa Cruz, CA, USA), rabbit α-phospho-Smad1/5/8 (pSmad); rabbit α-phospho-Akt(S) (pAkt) and rabbit α-Akt (Cell Signaling Technology); mouse α-flag (M2; Sigma); rat α-Netrin-1 (R&D Systems); rabbit α-ActRII (H65) and goat α-ActRIIB (N16) (Santa Cruz); mouse α-ActRIIB (abcam, Cambridge, MA USA); and mouse α-BMPRII (BD Transduction Laboratories, San Jose, CA, USA). We were unable to detect expression of ActRIIA using available ActRIIA-specific antibodies. However, an antibody that recognizes both ActRIIA and ActRIIB, α-ActRII (H65), preferentially detected the 70 kDa ActRIIA protein expressed in dI neurons (Figure 4A, lane 2). HRP- and fluorophore-conjugated secondary antibodies were purchased from Jackson ImmunoResearch Laboratories, West Grove, PA, USA. Cell culture reagents were: Ham's F12 medium, OptiMEM medium, penicillin/streptomycin/glutamine (P/S/G), penicillin/streptomycin (P/S), N2 supplement (Invitrogen, Carlsbad, CA, USA), FBS (Gemini BioProducts, West Sacramento, CA, USA), fibronectin (Sigma) and 45% glucose (Sigma). The mature region of mouse flag-tagged BMP7 was cloned into pMT23 as previously described [11,12]. Mouse Netrin-1.pMT23 was generously provided by Dr Thomas Jessell (Columbia University).

### Dissociated dI neuron culture

Embryonic day (E)13 rat dorsal spinal cord was dissected as previously described [3] in L15 medium (Sigma) and dissociated in 0.35% trypsin/0.2% glucose/PBS/P/S at 37°C for 15 minutes. Following trypsin digestion, the tissue was resuspended in culture medium (F12/10% FBS/P/S/G) and gently triturated. The single cell suspension was plated in culture medium onto poly-D-lysine/laminin-coated tissue culture dishes (western blot analysis) or 12 mm glass coverslips (immunolabeling) and incubated overnight at 37°C, 5% CO_2_. When required, dissociated dI neuron cultures were serum starved by incubation in non-supplemented F12 medium for 2 h at 37°C.

### Immunolabeling of dI neurons

Dissociated dI neuron cultures were fixed in 4% paraformaldehyde/PBS for 10 minutes, washed once in PBS, blocked and labeled with primary and Cy3-conjugated secondary antibodies diluted in 1% heat-inactivated goat serum or FBS/0.1% Triton X-100/PBS. For nuclear co labeling, DAPI (2.5 μg/ml; Sigma) was added with the secondary antibody. Coverslips were mounted onto glass microscope slides in Vectashield (Vector Laboratories, Inc., Burlingame, CA, USA).

### Phosphorylation assays

For phosphorylation assays, serum-starved dissociated dI neuron cultures were treated with either 4 mM HCl/0.1% BSA (control), BMP7 or BMP6, diluted in non-supplemented F12 medium at the indicated concentrations before immunolabeling with α-pSmad1/5/8 or α-pAkt or preparation of cell lysates for western blot analysis. BMP-treated [i] explants were labeled with α-phospho-Smad1/5/8.

### Western blot analysis

Whole cell lysates of dI neuron cultures were prepared using 1x lysis buffer (Cell Signaling Technology) supplemented with 1 mM PMSF. Samples were separated by SDS-PAGE (EZ-Run Gel Solution, Fisher Scientific, Pittsburgh, PA, USA) and transferred to nitrocellulose (Whatman, Clifton, NJ, USA). Nitrocellulose membranes were blocked in 5% nonfat milk/0.1% Tween 20/TBS (blocking buffer) and probed overnight with primary antibodies diluted in 5% BSA/0.1% Tween 20/TBS, except for the α-BMPRII and α-ActRIIB monoclonal antibodies, which were diluted in blocking buffer. Membranes were washed in TBST (0.1% Tween 20/TBS) and probed (1 hour) with HRP-conjugated secondary antibodies in blocking buffer. After washing in TBST, blots were developed using the Supersignal West Pico chemoluminescent substrate detection kit (Pierce, ThermoScientific, Rockland, IL, USA) and exposed to Kodak BioMax Light Film. For phosphorylation assays, membranes were washed in TBS, stripped for 30 minutes at 70°C in stripping buffer (62.5 mM Tris pH 6.8/2% SDS/0.1 M α-mercaptoethanol), washed in TBS and reprobed using antibodies that recognize total cellular Smad1/5/8 or Akt for normalization of the phosphorylated signals. The films were imaged using the Kodak Digital Science Image Station 440CF and densitometric analysis was performed using ImageJ v1.37 software (NIH).

### Growth cone collapse assay

Serum-starved dissociated dI neuron cultures were pre-incubated with DMSO (control), PI3K inhibitors (1 hour) or DM (30 minutes), diluted in non-supplemented F12 medium, and then stimulated with 50 ng/ml BMP7 or treated with 4 mM HCl/0.1% BSA (control) for 30 minutes. The cultures were fixed in pre-warmed 4% paraformaldehyde/0.5% gluteraldehyde/0.1 M phosphate buffer for 5 minutes, washed once in PBS, blocked in 1% heat-inactivated goat serum/0.1% Triton X-100/PBS and labeled with a mouse α-ERM IgM and a Cy3 goat α-mouse IgM secondary antibody. The growth cone area of neurons with axons greater than 10 μm was measured across two or three coverslips per condition for each experiment using ImageJ v1.37 software (NIH). Growth cone collapsing activity is presented as raw mean area or as the percentage decrease of growth cone area relative to control cultures.

### [i] explant assays

[i] explants were dissected from stage 10 chick embryos and cultured in collagen and immunolabeled as previously described [54,55]. BMPs or 4 mM HCl/0.1% BSA (control), with and without DMSO (control), DM or LY, were diluted in F12/N2 supplement/fibronectin/P/S) and incubated with the explants for 48 hours.

### [d] explant assays

E11 rat [d] explants were dissected, cultured and labeled as previously described [3]. For BMP7, BMP7:GDF7 or Netrin-1 expression, COS-1 cells were transfected with pMT23 expression constructs using Lipofectamine Reagent (Invitrogen), aggregated [56] and appended to [d] explants as described [3]. Inhibitors or DMSO (control), diluted in OptiMEM/P/S/G culture medium, were added at the beginning of the 36-h culture period. Explants were immunolabeled as described above. Quantification of Lhx2/9 induction, using ImageJ v1.37 software (NIH), was performed by measuring the integrated density (mean pixel intensity × area) of the BMP7-induced region of Lhx2/9^+ ^cells present in the explant. The angle of reorientation was measured as shown previously [3]. We observed similar induction of Lhx2/9 and axon orientation activity using COS-1 cell aggregates expressing either the BMP7 homodimer or the BMP7:GDF7 heterodimer [4] in [d] explant axon orientation assays with and without LY and present the combined data in Figures 5D and 6B.

### DM treatment

Dissociated dI neurons were pretreated with DM vehicle in solution or with 10 μM DM for 30 minutes and then incubated with BSA vehicle (control), or indicated BMPs, at 50 ng/ml for 30 minutes. In explants assays, DM was added at the time of culture, as were all other reagents. Dose response analysis was performed to determine an effective dose for blockade of type I BMP receptor kinase activity. Dissociated dI neurons, and [i] and [d] explants were treated with a range of DM concentrations (0 to 20 μM) for 30 minutes and then incubated with BSA vehicle (control) or BMPs. At 20 μM, neurons became unhealthy and were not further included in the study. At 0.1 and 1 μM there were no observable effects of DM treatment (not shown). DM was effective at 5 and 10 μM and these doses were used for all further experiments.

### LY and WM treatment

Dissociated dI neurons were pretreated with inhibitor vehicle solution, 50 μM LY or 100 nM WM for 1 h and stimulated with 50 ng/ml BMP7 or control for 30 minutes. For explants, 2.5 μM LY or 50 nM WM were added to explants at the time of culture.

### Imaging

Images of dI neuron dissociated cultures and explants were taken with a Zeiss AxioCam HR digital camera mounted on a Zeiss Axiovert 200 M fluorescence microscope. In addition, images of [i] and [d] explants were taken using a Zeiss LSM 5 confocal microscope and are presented here as confocal Z-stacks.

## Abbreviations

ActR: activin-like receptor; BMP: bone morphogenetic protein; BMPR: bone morphogenetic protein receptor; BSA: bovine serum albumin; [d] explants: explant of E11 rat dorsal spinal cord; DM: dorsomorphin; DMSO: dimethyl sulfoxide; dI dorsal spinal interneuron; E: embryonic day where E0.5 = 6 am on the day of plug; ERM: ezrin-radixin-moeisin; FBS: fetal bovine serum; HRP: horseradish peroxidase; [i] explant: explant of intermediate region of spinal cord of Hamburger Hamilton Stage 10 chick embryo; LIMK: LIM domain kinase; LY: LY294002; MAPK: mitogen-activated protein kinase; PBS: phosphate-buffered saline; PI3K: phosphoinositide-3-kinase; PKA: protein kinase A; P/S: penicillin/streptomycin; P/S/G: penicillin/streptomycin/glutamine; TAG-1: transient axonal glycoprotein; WM: wortmannin.

## Competing interests

The authors declare that they have no competing interests.

## Authors' contributions

The work was conceived, designed and planned by JP and JD in collaboration. JP performed all experiments described here. Analysis was performed by JP and JD. The manuscript was drafted by JP and JD and both authors approve the manuscript.

## Supplementary Material

Additional file 1 **Figure S1 - BMP7-mediated axon orientation is insensitive to changes in cAMP- and MAPK-dependent activity**. Histograms of the angle of reorientation in [d] explants co-cultured as in Figures 3D and 6A with pMT23- (dark gray bars) or BMP7-expressing (light gray bars) COS-1 cell aggregates incubated with or without inhibitors or activators as indicated. None of the reagents tested had any significant effect (Student's *t*-test) on control (pMT23) [d] explant co-cultures or on the repellent activity of BMP7 in [d] explant co-cultures. Results are expressed as the mean ± SEM for each condition. Angles of reorientation: control (pMT23) = -0.45 ± 1.8° (n = 11); BMP7 = 32.8 ± 1.6° (n = 35). PKA inhibitor (1 μM KT5720): pMT23 = -5.0 ± 2.1° (n = 3); BMP7 = 32.0 ± 2.9° (n = 7). Adenylate cyclase activator (4 μM forskolin): pMT23 = -1° (n = 1); BMP7 = 21.8 ± 4.9° (n = 4). Erk1/2 MAPK inhibitor (50 μM PD98059): pMT23 = -1.6 ± 2.3° (n = 3)); BMP7 = 28.0 ± 5.3° (n = 5). p38 MAPK inhibitor (10 μM SB203580): pMT23 = 1.3 ± 4.8° (n = 3); BMP7 = 31.0 ± 2.3° (n = 3). Click here for file
