# Supplementary figures and images for: Inductive specification and axonal orientation of spinal neurons mediated by divergent bone morphogenetic protein signaling pathways
Source: Neural Dev. 2011 Nov 15;6:36. doi: 10.1186/1749-8104-6-36 (PMC3227570; doi:10.1186/1749-8104-6-36)

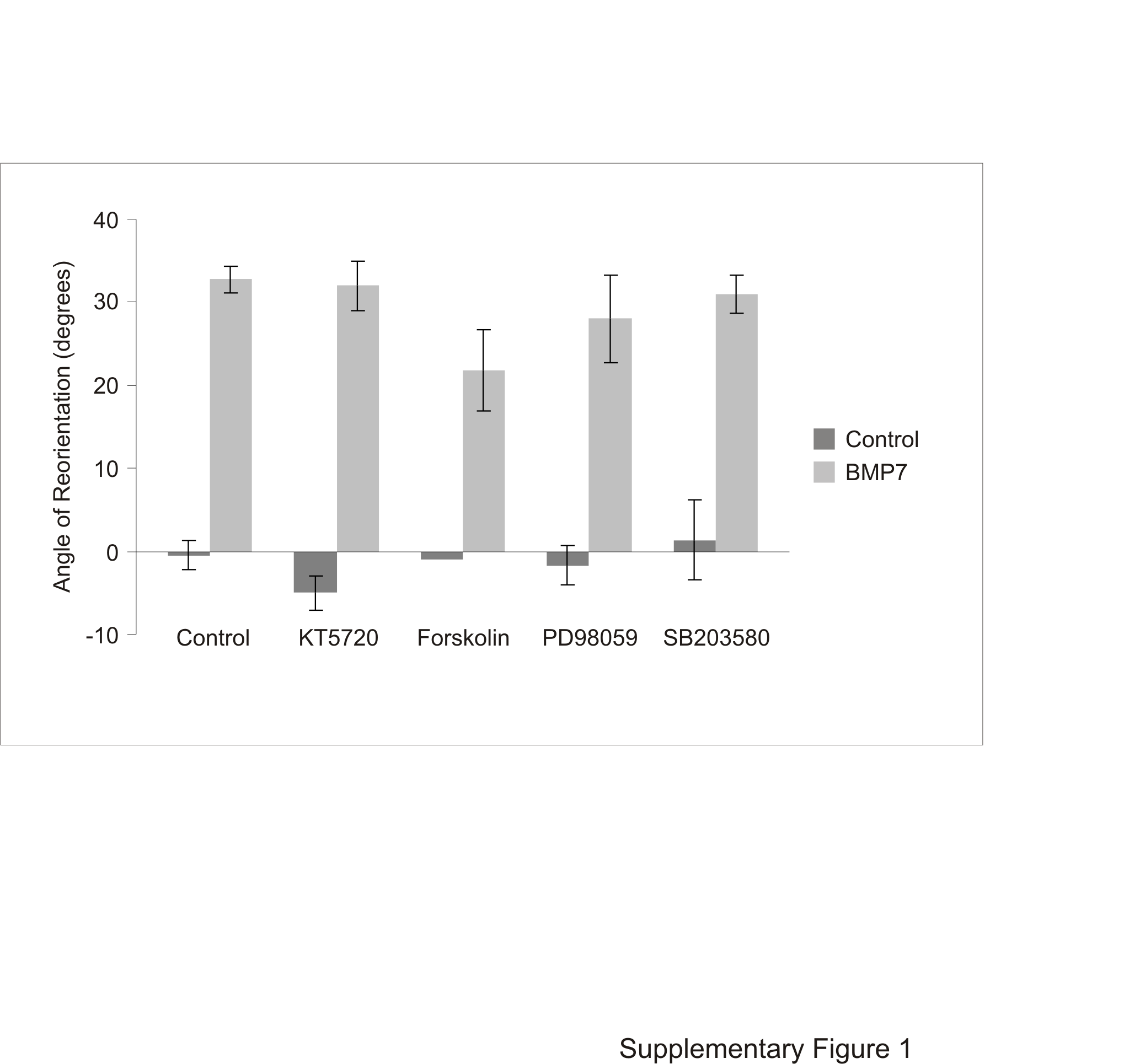

Supplement: Additional file 1 — Figure S1 - BMP7-mediated axon orientation is insensitive to changes in cAMP- and MAPK-dependent activity. Histograms of the angle of reorientation in [d] explants co-cultured as in Figures 3D and 6A with pMT23- (dark gray bars) or BMP7-expressing (light gray bars) COS-1 cell aggregates incubated with or without inhibitors or activators as indicated. None of the reagents tested had any significant effect (Student's t-test) on control (pMT23) [d] explant co-cultures or on the repellent activity of BMP7 in [d] explant co-cultures. Results are expressed as the mean ± SEM for each condition. Angles of reorientation: control (pMT23) = -0.45 ± 1.8° (n = 11); BMP7 = 32.8 ± 1.6° (n = 35). PKA inhibitor (1 μM KT5720): pMT23 = -5.0 ± 2.1° (n = 3); BMP7 = 32.0 ± 2.9° (n = 7). Adenylate cyclase activator (4 μM forskolin): pMT23 = -1° (n = 1); BMP7 = 21.8 ± 4.9° (n = 4). Erk1/2 MAPK inhibitor (50 μM PD98059): pMT23 = -1.6 ± 2.3° (n = 3)); BMP7 = 28.0 ± 5.3° (n = 5). p38 MAPK inhibitor (10 μM SB203580): pMT23 = 1.3 ± 4.8° (n = 3); BMP7 = 31.0 ± 2.3° (n = 3). [file 1749-8104-6-36-S1.TIFF]
